# Quantitative investigation of factors relevant to the T cell spot test for tuberculosis infection in active tuberculosis

**DOI:** 10.1186/s12879-019-4310-y

**Published:** 2019-07-29

**Authors:** Kui Li, Caiyong Yang, Zicheng Jiang, Shengxi Liu, Jun Liu, Chuanqi Fan, Tao Li, Xuemin Dong

**Affiliations:** 1 Department of Infectious Diseases, Ankang Central Hospital, 85 South Jinzhou Road, Ankang, 725000 Shaanxi Province China; 2 Department of Infectious Diseases, Ankang Central Hospital, Hubei University of Medicine, 30 South Renmin Road, Shiyan, 442000 Hubei Province China; 3 Laboratory of Molecular Pathology and Tuberculosis Diseases, Ankang Central Hospital, 85 South Jinzhou Road, Ankang, 725000 Shaanxi Province China

**Keywords:** Tuberculosis, T-SPOT.*TB*, IGRA, Diagnosis, Risk factors

## Abstract

**Background:**

Previous qualitative studies suggested that the false negative rate of the T cell spot test for tuberculosis infection (T-SPOT.*TB*) is associated with many risk factors in tuberculosis patients. However, more precise quantitative studies are lacking. The purpose of this study was to investigate the factors affecting quantified spot-forming cells (SFCs) to early secreted antigenic target 6 kDa (ESAT-6) or culture filtrate protein 10 kDa (CFP-10) in patients with active tuberculosis.

**Methods:**

We retrospectively analyzed the data of 360 patients who met the inclusion criteria. Using the SFCs to ESAT-6 or CFP-10 levels as dependent variables, variables with statistical significance in the univariate analysis were subjected to optimal scaling regression analysis. The combination of ESAT-6 and CFP-10 (i.e., T-SPOT.*TB*) was analyzed by the exact logistic regression model.

**Results:**

The results showed that the SFCs to ESAT-6 regression model had statistical significance (*P* < 0.001) and that previous treatment and CD4+ and platelet counts were its independent risk factors (all *P* < 0.05). Their importance levels were 0.095, 0.596 and 0.100, respectively, with a total of 0.791. The SFCs to CFP-10 regression model also had statistical significance (*P* < 0.001); platelet distribution width and alpha-2 globulin were its independent risk factors (all *P* < 0.05). Their importance levels were 0.287 and 0.247, respectively, with a total of 0.534. The quantification graph showed that quantified SFCs to ESAT-6 or CFP-10 grading had a linear correlation with risk factors. Albumin-globulin ratio, CD4+ and CD8+ were independent risk factors for false negative T-SPOT.*TB* (all *P* < 0.05).

**Conclusions:**

In T-SPOT.*TB*-assisted diagnosis of patients with active tuberculosis, previous treatment, decreased CD4+ and platelet count might lead to the decreased SFCs to ESAT-6, decreased alpha-2 globulin and high platelet distribution width might lead to the decreased SFCs to CFP-10, decreased albumin-globulin ratio, CD4+ and CD8+ might lead to an increase in the false negative rate of the T-SPOT.*TB*.

**Electronic supplementary material:**

The online version of this article (10.1186/s12879-019-4310-y) contains supplementary material, which is available to authorized users.

## Background

The interferon-gamma release assay (IGRA) represents one of the most important advances in the immunodiagnosis of *Mycobacterium tuberculosis* (MTB) infection in the past two decades. As a new adjuvant method for the diagnosis of MTB infection, IGRA has been widely applied and accepted clinically. In principle, IGRA determines whether the subject is infected with MTB by examination of the levels of released γ-interferon (IFN-γ) after stimulation of whole blood or peripheral blood mononuclear cells (PBMCs) with MTB-specific antigen. This test is not affected by Bacillus Calmette-Guerin (BCG) vaccination [1], a feature that is very beneficial in countries such as China in which general BCG vaccination is practiced. Currently, the T cell spot test for tuberculosis infection (T-SPOT.*TB*) is the main IGRA test method; it provides intuitive and reproducible results and quantitatively reflects the number of IFN-γ secreting cells in preparations of PBMCs [2].

IGRA still has a certain false negative rate among patients with tuberculosis (TB). Previous studies reported that negative bacteria in sputum [3–5], hypoproteinemia [6–8], combined HIV infection [4, 7, 9], anti-TB treatment [10, 11], medical history [8, 12], anemia [6, 13], diabetes [14], parasitic infections [13], noncavitary lesions in the lung [5], fall and winter seasons [15], increased human leukocyte antigen DRB1–0701 allele [16] and non-Hispanic white or Asian ethnicity [6, 9] are risk factors for false negative IGRA. The association between IGRA and age [3, 7–9, 12, 13, 15–18], body mass index (BMI) [12, 16] and reduced lymphocyte levels [3, 5, 7, 8, 17, 19, 20] is inconsistent among previous studies. Some of these studies were qualitative studies with small samples and did not consider the possibility that different antigen risk factors might have confounded or biased the results. In addition, there is a lack of quantitative examination of the association between spot-forming cells (SFCs) within PBMCs and clinical and laboratory characteristics. As a result, further research is urgently needed. Our main objective based on population analysis is to identify factors associated with different antigen quantification results of T-SPOT.*TB* in active TB (ATB), with the goal of providing more reference evidence for the accurate application of T-SPOT.*TB*.

## Methods

### Study populations

**Fig. 1:**
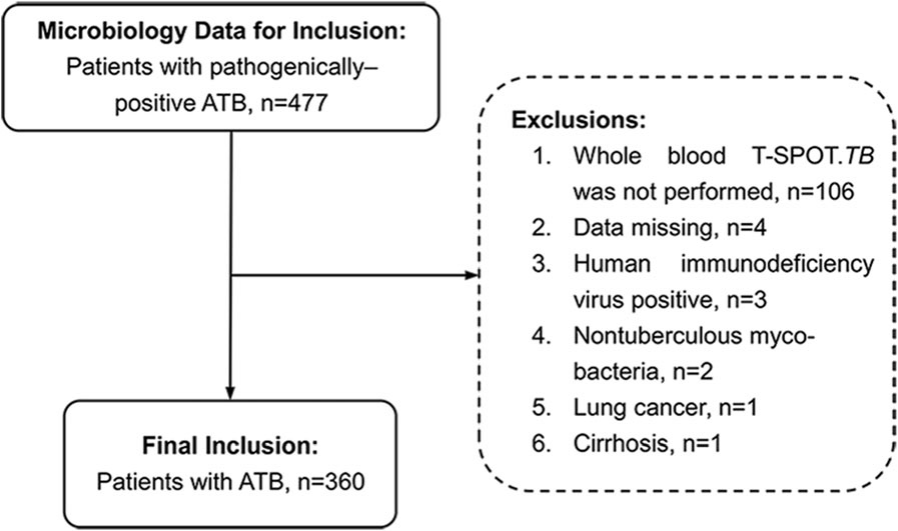
Description of the sample. *ATB* Active tuberculosis

This retrospective study analyzed 360 pathogenically positive TB patients who were hospitalized in Ankang Central Hospital, Shaanxi, China, and the data were accessed between October 1, 2016 and June 30, 2018. We collected data on 29 variables associated with five aspects, including the patients’ general status, bacteriology, imaging, routine examination, protein electrophoresis and immunology. The subject screening process is illustrated in (Fig. 1).

This study was approved by the ethics committee of Ankang Central Hospital (ECACH-2016011). Informed consent was not required because the study did not put the patients at risk. Prior to analysis, the patients’ identities were protected by anonymity and by the use of codes.

### Inclusion and exclusion criteria

The inclusion criteria were as follows [21]: (1) the MTB-specific deoxyribonucleic acid or ribonucleic acid test was positive, and patients had suspected symptoms of TB; (2) patients had one sputum smear that was positive for acid-fast staining or positive sputum culture, and thoracic imaging showed lesions conforming to ATB and/or suspected symptoms of TB; (3) patients were subjected to the T-SPOT.*TB* test, and the results were accessible.

The exclusion criteria were as follows: (1) patients who were taking immunosuppressants; (2) patients who did not undergo IGRA examination; (3) patients who were positive for HIV infection; (4) patients with nontuberculous mycobacteria infection; (5) patients with combined cirrhosis; (6) patients with combined tumor.

### T-SPOT.*TB* assay

The T-SPOT.*TB* diagnosis kit was provided by Shanghai Fosun Long March Medical Science Co., LTD (Oxford Immunotec Ltd., United Kingdom). Specimens were examined within 2 h of collection at room temperature (18–25 °C) in a sterile environment. (1) RPMI 1640 medium was mixed with an equal volume of a whole blood sample. (2) Three milliliters of Ficoll human lymphocyte isolation solution was added to the first centrifuge tube; 6–9 ml of diluted whole blood was then slowly added to the solution, and the tube was centrifuged at 1000 x g for 22 min. (3) Approximately 5 ml of the layer containing PBMCs was transferred to a second centrifuge tube, and the mixture was brought to a volume of 10 ml with RPMI 1640; the tube was then inverted and centrifuged at 600 x g for 7 min. (4) The supernatant was discarded, the pellet was resuspended in 1 ml of medium, and RPMI 1640 was added to bring the final volume to 10 ml. After mixing by inverting the tube, the sample was centrifuged at 350 x g for 7 min. (5) The supernatant was discarded, and the pellet was resuspended in AIM-V medium at a density of 2.5 × 10^6^ cells/ml. (6) Fifty microliters of negative control (ALM-V), antigen A (early secreted antigenic target 6 kDa, ESAT-6), antigen B (culture filtrate protein 10 kDa, CFP-10) and positive control (PHA) were added sequentially to an IFN-γ antibody-coated microplate; 100 μl of diluted cells were then added to each well, and the plate was placed in a CO_2_ incubator (37 °C, 5% CO_2_) for 20 h. (7) After removal of the medium, each well was washed four times with 200 μl of PBS and incubated with 50 μl of secondary antibody working solution (1200) for 1 h at 2–8 °C. (8) After removal of the secondary antibody, each well was washed four times with PBS, 50 μl of substrate chromogen solution was added to each well, and the plate was allowed to stand for 10 min in the dark. The reaction was terminated by washing the plate with distilled water. (9) SFCs were automatically counted using a plate reader (ES-15, Shanghai Fosun Long March Medical Science Co., Ltd., Shanghai, China). When the counts could not be determined using the plate reader, the SFCs were manually counted using a microscope. The results were recorded and interpreted as recommended by the kit manufacturer: ESAT-6 or CFP-10 ≥ 6 SFCs was regarded as T-SPOT.*TB* positive. T-SPOT.*TB* was negative if both ESAT-6 and CFP-10 were < 6 SFCs. The test results were uncertain if there were > 10 SFCs in the blank control well or < 20 SFCs in the positive control well. In cases of uncertain results, blood samples were taken again for another test.

### Diagnosis of TB

Sputum or body fluid specimens were first examined using Auramine O fluorescence staining (KRJ/TTR500 automatic smear staining machine, Xiangyang Courager Medical Apparatus, Xiangyang, China) and were then subjected to MTB nucleic acid amplification (RNA constant temperature amplification, Roche LightCycler 480 II Real-time PCR Cycler; Rendu Biotechnology [Shanghai, China]; PCR-fluorescence method, Applied Biosystems 7500 Real-Time PCR System; DAAN Gene of Sun Yat-sen University [Guangzhou, China]) and rapid drug tolerance gene analysis (DNA microarray chip method; CapitalBio Corporation, Chengdu, China). Finally, the specimens were subjected to MTB culture (Roche fixed culture test), bacterial species identification and drug sensitivity testing (Ratio methods, BaSo Diagnostics INC., ZHUHAI). Using the Roche solid culture as the standard and no repeated counting, bacterial load was reported in accordance with the “*Diagnostic Criteria and Principles of Management of Infectious Pulmonary Tuberculosis*” [21] (see Additional file 1). The laboratory meets the national P3 requirements and accepts the quality control and management of the national reference laboratory. All operators received special training in the testing methods and in operation of the apparatus.

### Measurement of other variables

BMI was calculated by dividing the individual’s body weight (kg) by the square of his or her height (m). Body weight and height were measured at the time of admission. Behavioral risk factors (such as smoking and exposure to dust) and medical history were collected by physicians. The diagnostic criteria for diabetes were random blood glucose ≥11.1 mmol/L or fasting blood glucose ≥7.0 mmol/L [22]. The severity of lung imaging was graded according to the criteria set by the National Tuberculosis Association of America [23] (see Additional file 2). Whole blood cell counts were determined using a Sysmex XN-9000 automatic blood fluid analyzer and supporting reagents. Serum protein electrophoresis was performed with the Sebia Capillarys 2 Flex Piercing detection platform and supporting reagents (Pare Technologique Leonard de Vinci, CP8010-Lisses 91,008 EVRY CEDEX, France). Lymphocyte subsets were detected using the Mindray flow cytometer BriCyte E6 and a kit from BD.

### Statistical analysis

The data were analyzed using SPSS software (version 22.0, IBM Corp., Armonk, NY, USA). Non-normally distributed data were expressed as medians and interquartile ranges. Wilcoxon’s rank sum test was used to compare two-sample dichotomous variables. The rank data and measurement data were analyzed using the Spearman relationship test, and the statistically significant variables were subjected to optimal scaling regression analysis. The false negative rate of T-SPOT.*TB* was analyzed by the exact logistic regression model of R software (version 3.4.4; https://www.r-project.org). Tolerance ≥0.1 is considered as collinearity between variables. The missing values of the independent variables were replaced by the series mean. Adobe Photoshop CS6 software (version 13.0 × 32, Adobe Systems Incorporated, San Jose, CA, USA) was used for graphing. *P* < 0.05 was considered statistically significant.

## Results

We recruited 360 patients with an average age of 53.00 years (ranging 36.00–66.00 year), of whom 78.06% were male (281/360). The false negative rates of ESAT-6, CFP-10 and T-SPOT.*TB* were 8.61% (31/360), 10.00 (36/360) and 3.33% (12/360), respectively. The true positive and false negative rates for T-SPOT.*TB* test in pulmonary and extra pulmonary TB groups are shown in Additional file 3.

Univariate analysis showed that there was statistical significance in the yes or no of previously treated cases with SFCs to ESAT-6 (*P* < 0.05). CD4+, CD8+, platelet (PLT), prealbumin, albumin, albumin-globulin ratio and beta-1 globulin were positively associated with SFCs to ESAT-6 (all *P* < 0.05). Alpha-1 globulin was negatively associated with SFCs to ESAT-6 (*P* < 0.05). CD4+, CD8+, PLT, prealbumin, albumin and alpha-2 globulin were positively associated with SFCs to CFP-10 (all *P* < 0.05). Age and platelet distribution width (PDW) were negatively associated with SFCs to CFP-10 (all *P* < 0.05). The associations with other independent variables, including gender, sputum bacterial load and BMI, were not statistically significant (all *P* > 0.05) (Tables 1 and 2). The differences in age and gender were not statistically significant between negative and positive T-SPOT.*TB* (*P* > 0.05), and prealbumin, albumin, albumin-globulin ratio, CD4+ and CD8+ differed significantly between the two groups (*P* < 0.05) (see Additional files 4 and 5).

**Table 1:** Univariate analysis of SFCs to ESAT-6 or CFP-10 (nominal and ordinal variables)

Variable	N	SFCs to ESAT-6			SFCs to CFP-10		
		SFCs/250000PBMCs	Test value	*P* value	SFCs/250000PBMCs	Test value	*P* value
Total	360	69.0 (24.3–148.3)			83.5 (23.0–218.3)		
Sex							
Female	79	87.0 (42.0–155.0)			97.0 (42.0–200.0)		
Male	281	66.0 (22.5–134.5)	Z = 12,681.5	0.053	82.0 (21.0–227.0)	Z = 11,627.5	0.518
Smoking^a^							
No	147	71.0 (37.0–143.0)			92.0 (29.0–234.0)		
Yes	213	66.0 (20.0–150.0)	Z = 14,353.0	0.180	82.0 (21.5–205.5)	Z = 15,138.0	0.594
Dust exposure^a^							
No	257	69.0 (27.0–146.5)			82.0 (22.0–200.0)		
Yes	103	59.0 (19.0–150.0)	Z = 12,438.5	0.372	85.0 (28.0–237.0)	Z = 13,966.0	0.413
Previously treated cases							
No^b^	257	74.0 (28.5–150.0)			91.0 (23.0–235.5)		
Yes^c^	103	54.0 (13.0–97.0)	Z = 10,233.0	0.001	65.0 (23.0–200.0)	Z = 12,289.5	0.289
Cavitation							
No	137	72.0 (26.5–144.0)			91.0 (18.0–200.0)		
Yes	223	67.0 (24.0–150.0)	Z = 15,093.0	0.849	82.0 (24.0–257.0)	Z = 15,772.5	0.604
Extrapulmonary tuberculosis							
No	297	69.0 (24.0–150.0)			82.0 (23.0–200.0)		
Yes	63	66.0 (25.0–114.0)	Z = 8997.5	0.633	121.0 (24.0–300.0)	Z = 9996.5	0.393
Diabetes mellitus							
No	330	70.0 (24.8–150.0)			87.0 (24.0–220.3)		
Yes	30	38.5 (22.0–89.5)	Z = 3920.0	0.059	49.5 (7.8–216.0)	Z = 4321.0	0.249
Drug-resistant tuberculosis							
No	327	69.0 (25.0–150.0)			85.0 (23.0–220.0)		
Yes	33	65.0 (15.5–134.5)	Z = 5181.0	0.707	71.0 (25.0–199.0)	Z = 5409.0	0.981
Stage^d^							
Minimal/Mild	2	77.5 (0.0–77.50)			85.5 (22.0–85.5)		
Moderate	143	78.0 (38.0–150.0)			97.0 (41.0–264.0)		
Advanced	215	64.0 (22.0–126.0)	SRCC = -0.095	0.071	82.0 (15.0–211.0)	SRCC = 0.000	0.995
Grade^e^							
DNA/RNA positive	80	68.0 (27.3–99.5)			84.0 (22.5–251.8)		
The number of colony	24	104.0 (51.8–203.5)			132.5 (24.8–262.5)		
1+	102	67.0 (21.8–150.0)			77.0 (22.8–205.0)		
2+	50	59.5 (25.5–142.3)			81.0 (21.3–291.8)		
3+	70	69.0 (24.5–150.0)			86.0 (26.8–200.0)		
4+	34	65.5 (30.3–126.8)	SRCC = -0.075	0.158	59.5 (19.3–154.8)	SRCC = -0.029	0.587

Data are presented as N, median (interquartile range). There were no missing values

^a^: Smoking or dust exposure for at least 3 months before a diagnosis of pulmonary tuberculosis

^b^: New cases are defined as not starting anti-TB treatment or being on anti-TB treatment for <1 month

^c^: Previously treated cases are defined as those anti-TB treated ≥1 month in the past

^d^: The staging standard of National Tuberculosis Association of the United States of America

^e^: Smear grading before treatment

*CFP-10* Culture filtrate protein 10 kDa, *DNA* Deoxyribonucleic acid, *ESAT-6* Early secreted antigenic target 6 kDa, *PBMCs* Peripheral blood mononuclear cells, *RNA* Ribonucleic acid, *SFCs* Spot-forming cells, *SRCC* Spearman’s rho correlation coefficient, *Z* Wilcoxon’s rank sum test

**Table 2:** Univariate analysis of SFCs to ESAT-6 or CFP-10 (numeric variables)

Variable	Missing data		Median (interquartile range)^a^	SFCs to ESAT-6		SFCs to CFP-10	
	N	(%)		SRCC	*P* value	SRCC	*P* value
Age, years	0	(00.0)	53.0 (36.0–66.0)	− 0.102	0.054	− 0.107^b^	0.042
Body mass index, kg/m^2^	6	(1.7)	19.1 (17.6–21.2)	0.030	0.565	0.013	0.801
Duration of symptoms, days	0	(00.0)	90.0 (30.0–496.3)	− 0.073	0.165	0.025	0.630
Hemoglobin, g/L	0	(00.0)	118.0 (105.0–134.8)	0.034	0.518	0.016	0.765
Platelet, ×10^9^/L,	0	(00.0)	258.0 (191.0–320.8)	0.173^c^	0.001	0.186^c^	<0.001
Platelet distribution width, fL	0	(00.0)	14.0 (11.7–16.2)	−0.090	0.089	−0.116^b^	0.027
Erythrocyte sedimentation rate, mm/H	32	(8.9)	49.9 (28.3–69.0)	−0.070	0.184	−0.028	0.594
Prealbumin, mg/L	39	(10.8)	143.6 (84.3–184.8)	0.187^c^	<0.001	0.104^b^	0.050
Albumin, g/L	1	(0.3)	31.9 (27.6–36.5)	0.159^c^	0.002	0.108^b^	0.040
Globulin, g/L	5	(1.4)	32.6 (28.9–36.1)	0.014	0.795	0.049	0.356
Albumin-globulin ratio	5	(1.4)	1.0 (0.8–1.2)	0.105^b^	0.047	0.054	0.303
Alpha-1 globulin, g/L	40	(11.1)	4.9 (3.7–5.6)	−0.122^b^	0.020	−0.077	0.146
Alpha-2 globulin, g/L	40	(11.1)	7.6 (6.5–8.6)	0.091	0.085	0.141^c^	0.007
Beta-1 globulin, g/L	40	(11.1)	3.7 (3.3–4.1)	0.111^b^	0.036	0.058	0.270
Beta-2 globulin, g/L	40	(11.1)	3.7 (3.2–4.1)	0.034	0.514	0.063	0.233
Gamma globulin, g/L	40	(11.1)	13.0 (10.4–14.7)	0.012	0.823	0.014	0.797
CD4+ T lymphocytes, cells/μL	89	(24.7)	302.0 (203.0–445.0)	0.391^c^	<0.001	0.230^c^	<0.001
CD8+ T lymphocytes, cells/μL	89	(24.7)	253.0 (164.0–379.0)	0.291^c^	<0.001	0.190^c^	<0.001
CD4+ to CD8+ ratio	89	(24.7)	1.2 (0.9–1.7)	0.049	0.354	−0.042	0.425

^a^: Data are presented as the value of variable

^b^: Correlation is significant at the 0.05 level (2-tailed)

^c^: Correlation is significant at the 0.01 level (2-tailed)

*CFP-10* Culture filtrate protein 10 kDa, *ESAT-6* Early secreted antigenic target 6 kDa, *SFCs* Spot-forming cells, *SRCC* Spearman’s rho correlation coefficient

Optimal scaling regression analysis showed that the regression models were statistically significant (SFCs to ESAT-6, adjusted R-squared = 0.147, F = 7.884, *P* < 0.001; SFCs to CFP-10, adjusted R-squared = 0.061, F = 3.890, *P* < 0.001). Analysis of all the included factors showed that previously treated cases, CD4+ and PLT were significantly associated with SFCs to ESAT-6; their importance levels were 0.095, 0.596 and 0.100, respectively, with a total of 0.791. PDW and alpha-2 globulin were also significantly associated with SFCs to CFP-10; their importance levels were 0.287 and 0.247, respectively, with a total of 0.534. The tolerances of the included factors in the two models were > 0.1, indicating that there was no serious collinearity among the factors and that the regression models were reliable (Table 3).

**Table 3:** Optimal scale regression analysis of SFCs to ESAT-6 or CFP-10

Variable	Beta	Standardized	df	F	Sig.	Tolerance		Importance
		coefficients Std.Error				After transformation	Before transformation	
SFCs to ESAT-6^a^								
Previously treated cases	0.103	0.046	1	5.110	0.024	0.948	0.948	0.095
Platelet, ×10^9^/L,	0.115	0.054	1	4.471	0.035	0.818	0.818	0.100
Prealbumin, mg/L	0.043	0.072	1	0.366	0.545	0.492	0.492	0.040
Albumin, g/L	0.080	0.094	1	0.738	0.391	0.253	0.253	0.076
Albumin-globulin ratio	−0.075	0.082	1	0.819	0.366	0.274	0.274	−0.039
Alpha-1 globulin, g/L	−0.092	0.056	1	2.630	0.106	0.725	0.725	0.072
Beta-1 globulin, g/L	−0.016	0.062	1	0.067	0.796	0.617	0.617	−0.008
CD4+ T lymphocytes, cells/μL	0.277	0.075	1	13.539	0.000	0.598	0.598	0.596
CD8+ T lymphocytes, cells/μL	0.048	0.063	1	0.588	0.444	0.658	0.658	0.069
SFCs to CFP-10^b^								
Age, years	−0.022	0.057	1	0.153	0.696	0.867	0.867	0.018
Platelet, ×10^9^/L,	0.027	0.068	1	0.161	0.688	0.634	0.634	0.047
Platelet distribution width, fL	−0.149	0.058	1	6.549	0.011	0.701	0.701	0.287
Prealbumin, mg/L	−0.032	0.076	1	0.179	0.672	0.539	0.539	−0.021
Albumin, g/L	0.118	0.070	1	2.830	0.093	0.488	0.488	0.131
Alpha-2 globulin, g/L	0.133	0.049	1	7.464	0.007	0.917	0.917	0.247
CD4+ T lymphocytes, cells/μL	0.115	0.071	1	2.648	0.105	0.608	0.608	0.236
CD8+ T lymphocytes, cells/μL	0.031	0.063	1	0.245	0.621	0.652	0.652	0.055

^a^: R Square = 0.169; Adjusted R Square = 0.147; F = 7.884; *P*<0.001

^b^: R Square = 0.081; Adjusted R Square = 0.061; F = 3.890; *P*<0.001

*CFP-10* Culture filtrate protein 10 kDa, *ESAT-6* Early secreted antigenic target 6 kDa, *SFCs* Spot-forming cells

**Fig. 2:**
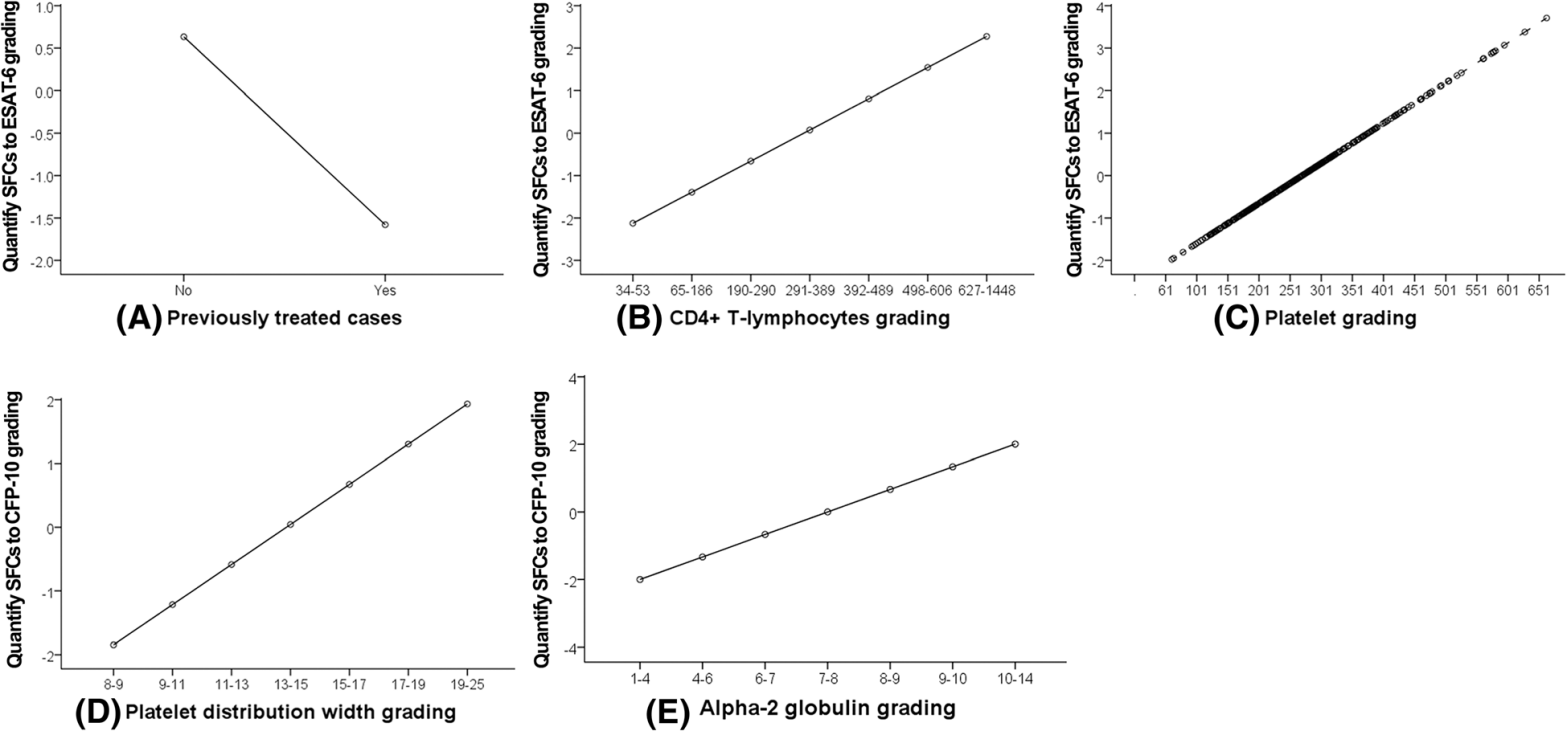
Conversion graph between the quantified values of the independent and dependent variables. The abscissa (x-axis) represents the value before variable conversion, and the ordinate (y-axis) represents the quantified SFCs to ESAT-6 or CFP-10 grading. **a** Patients with a history of previous treatment showed decreased SFCs to ESAT-6 grading. **b** Quantified SFCs to ESAT-6 grading increased with an increase in the number of CD4+ T lymphocytes. **c** Quantified SFCs to ESAT-6 grading increased with an increase in the number of PLT. **d** Quantified SFCs to CFP-10 grading increased with an increase in PDW grading. **e** Quantified SFCs to CFP-10 grading increased with an increase in alpha-2 globulin grading. *CFP-10* Culture filtrate protein 10 kDa, *ESAT-6* Early secreted antigenic target 6 kDa, *SFCs* Spot-forming cells

The quantification graph after variable conversion showed the following. (1) The quantified SFCs to ESAT-6 grading of previously treated cases was lower than that of new cases (Fig. 2a). (2) The quantified SFCs to ESAT-6 grading increased as CD4+ and PLT increased (Fig. 2b and c). (3) The quantified SFCs to CFP-10 grading increased as PDW and alpha-2 globulin increased (Fig. 2d and e).

Exact logistic regression analysis showed that albumin-globulin ratio, CD4+ and CD8+ were independent risk factors for false negative T-SPOT.*TB* (all *P* < 0.05) (Table 4).

**Table 4:** Exact logistic regression analysis of false negative T-SPOT.*TB*

Variable	Estimate	*P* value	Standard error for *p*-value	Simulation sample size for Monte Carlo	95% confidence intervals for parameters	
					Lower	Upper
Joint^a^	NA	0.873	0.015	8300	NA	NA
Prealbumin	−0.126	0.740	0.037	1205	−1.525	1.359
Albumin	0.813	0.133	0.018	3414	−0.252	2.585
Albumin-globulin ratio	0.880	0.035	0.009	4866	0.008	2.262
CD4+ T lymphocytes	0.551	0.028	0.008	3491	0.059	1.196
CD8+ T lymphocytes	0.498	0.035	0.010	2724	−0.033	1.324

^a^: It represents a test that all the parameters for the exact statement are simultaneously equal to zero in addition to the tests of the individual parameters

*NA* Not applicable

## Discussion

In ATB, the number of MTB increases and the number as well as amount of secretion of activated MTB-specific effector cells increases with it. Relevant studies have also shown that the level of SFCs differs between latent tuberculosis infection (LTBI) and ATB [24], and between before and after anti-tuberculosis treatment [25, 26], which indicates that it could serve as a complementary approach to make a distinction between ATB and LTBI and to predict the progress of LTBI into ATB. Seeing that the existence of T-SPOT.*TB* false negative was related to SFCs and tuberculosis activity level, the study on factors influencing quantitative changes in SFCs would provide reference to the improvement of T-SPOT.*TB* detection sensitivity and the development of its field of application.

In this study, the observed false negative rate of T-SPOT.*TB* was lower than the T-SPOT.*TB* false negative rate of 6.74% (61/905) reported in a previous study [27]. This discrepancy may be due to inconsistencies in case inclusion criteria and to the existence of different comorbidities. Children or patients who are taking immunosuppressants may impact the uncertain outcomes. In the current study, children (0–14 years old) accounted for 0.83% (3/360), and patients who were using immunosuppressants were excluded, so they had minimal impact on our results.

This study showed that the SFCs to ESAT-6 of previously treated cases was significantly lower than that of new cases. The causes may include the following. (1) As the disease progresses, the cellular immune function of the body further declines, and the corresponding immune reactions are gradually weakened. Cell activation or immunosuppression is further aggravated, and this aggravation weakens the immune response of the T lymphocytes to MTB and leads to a reduced number of cells releasing IFN-γ. (2) MTB may directly and continuously downregulate helper T cells (Th1), leading to reduced function of Th1 in secreting IFN-γ [28]. (3) Following anti-TB treatment, the MTB is gradually cleared from the body. However, the TB antigen-specific effector T cells are also reduced in number or even disappear, which may lead to a decline in SFCs to ESAT-6. This outcome is consistent with a previous study [29].

In this study, 17.50% (63/360) of the patients had extra pulmonary tuberculosis, and we did not find an association between pulmonary tuberculosis and extrapulmonary tuberculosis in the T-SPOT.*TB* results (see Additional file 3). Additionally, another study reported increased activation frequency of regulatory T cells and T lymphocytes in patients with extrapulmonary tuberculosis [30], indicating that this relationship cannot be replaced by each other in the complex immune regulatory network of the body.

Age and diabetes had a marginally significant effect in quantitative analysis, while there was no effect in qualitative analysis, which is not consistent with previous studies [14–18]. This might be because we included parameters that are more associated with T-SPOT.*TB*, age and diabetes were excluded due to their limited contribution.

BMI is an alternative indicator of body’s nutritional status. BMI had no effect in our study whether it was quantitative, qualitative or two extreme cases (BMI < 16.00 kg/m^2^ accounted for 10.73% (38/354) and BMI ≥ 25.00 kg/m^2^ accounted for 4.00% (14/354) (see Additional file 6)). Unfortunately, we lacked data for BMI ≥ 25.00 kg/m^2^ in the results of false negative T-SPOT.*TB*. This finding is inconsistent with some previous studies [12, 16], which may be related to variable type, BMI data distribution and the different location and social environment of subjects.

It was found in this study that SFCs to ESAT-6 or CFP-10 were positively correlated with the number of PLT, but negatively correlated with PDW. Finally, this correlation only maintained between PLT and SFCs to ESAT-6, PDW and SFCs to CFP-10 in the multivariate analysis. PLT might stimulate T lymphocyte adhesion by secreting RANTES (regulated on activation, normal T-cell expressed and secreted, also known as CCL5), a key chemical inducer, so as to regulate T lymphocytes [31, 32]. PDW is a parameter that reflects the variation in PLT volume, The α-granules [33, 34] and P-selectin involved in immuno regulation in PLT were the main factors affecting PDW. It is possible that the number of PLT with small volumes and lacking P-selectin leads to higher bacterial load in the lungs [35], when the body is stimulated by the more MTB antigen, it produced more SFCs to CFP-10. PLT and PDW showed the trend above in the T-SPOT.*TB* false negative analysis, but there was no statistical significance, which was inconsistent with the research of Kim et al. [36], and it might be related to the small number of false negative cases in this study.

The preliminary studies showed that, the decrease of albumin [37] and the increase of alpha-2 globulin [38] could be seen in the patients with pulmonary tuberculosis, which just reduced the albumin-globulin ratio, thereby affecting the body’s immune function, and possible mechanisms might be: (1) Malnutrition may impair the functions of antigen presenting cells [39] and inhibit glucose metabolism-dependent T cell activation [40, 41]. (2) Alpha-2 globulin mainly includes haptoglobin, ceruloplasmin, and alpha-2 macroglobulin. Haptoglobin and ceruloplasmin increased as acute phase reaction proteins; alpha-2 macroglobulin increased in a compensatory manner to maintain plasma colloid osmotic pressure due to hypoalbuminemia. Nevertheless, in the final analysis, it was alpha-2 globulin that affected SFCs to CFP-10, rather than albumin, which could not be fully explained by the mechanism of compensatory increase; albumin-globulin ratio showed more potential as an indicator of nutritional status and chronic infection, and all of these problems deserve further studies.

It was found in the multivariate analysis that, CD4+ was positively correlated with SFCs to ESAT-6, and CD4+ and CD8+ were positively correlated with false negative T-SPOT.*TB*; while, CD8+ showed no correlation with SFCs to ESAT-6 or CFP-10. The quantification graph following variable conversion showed that increased CD4+ counts were associated with higher SFCs to ESAT-6 grading, and the importance level was relatively high, suggesting that CD4+ count is a main factor affecting SFCs to ESAT-6. It also suggests that CD4+ cells may play a more important role in MTB infection than CD8+ cells [42]. This finding is inconsistent with a previous study in which the immune status of the subject had minimal effect on IGRA-ELISPOT test results [43] but is consistent with the study of Zhang et al. [5]. This may be related to one or more of the following factors. (1) When a patient infected with TB is immunized, large numbers of lymphocytes accumulate at the lesion, leading to a decrease in the number of lymphocytes in the peripheral circulation and a reduction in the number of active T lymphocytes; this eventually leads to reduced numbers of SFCs in the T-SPOT.*TB* test. (2) SFCs to ESAT-6 is more closely associated with the transcription and expression of IFN-γ by T lymphocytes [44]. (3) During immunization, CD4+ T cells preferentially release interferon, whereas CD8+ T cells preferentially lyse antigen-presenting cells [45]. Further study and analysis are required to determine the importance of changes in the number of CD4+ T lymphocytes in this process.

The limitations of this study are as follows. First, the amount of information available for interpreting T-SPOT.*TB* variation was low, and other unknown factors remain to be investigated. Second, unlike demographic, clinical and laboratory characteristics, the genetic causes of false negative T-SPOT.*TB* results cannot be studied. Finally, there were limited cases with false negative T-SPOT.*TB,* so that its results were less reliable and more samples are needed for further studies. However, the quantitative and qualitative analysis in this study showed that, CD4+ and serum protein affected the detection results of T-SPOT.*TB* in ATB patients, and the correlation between PDW, alpha-2 globulin and SFCs to CFP-10 was reported for the first time.

## Conclusions

In T-SPOT.*TB*-assisted diagnosis of patients with ATB, previous treatment, CD4+ and PLT are factors affecting SFCs to ESAT-6, and PDW and alpha-2 globulin are factors affecting SFCs to CFP-10. Albumin-globulin ratio, CD4+ and CD8+ are independent risk factors for false negative T-SPOT.*TB*. The influence factors for these three are different. Detailed assessment of these factors may help us accurately understand the diagnosis of TB using T-SPOT.*TB.*

## Additional files

Additional file 1: Smear grading report standard^a^. (DOC 31 kb)

Additional file 2: Thoracic CT scan image classification criteria^a^. (DOC 32 kb)

Additional file 3: T-SPOT.*TB* results in pulmonary tuberculosis and extrapulmonary tuberculosis patients (DOC 51 kb)

Additional file 4: Comparison of different influencing factors among tuberculosis patients with negative and positive T-SPOT.*TB* (dichotomy and grade variables) (DOC 105 kb)

Additional file 5: Comparison of different influencing factors among tuberculosis patients with negative and positive T-SPOT.*TB* (numeric variables) (DOC 80 kb)

Additional file 6: Results of T-SPOT.*TB* between the two extreme BMI (DOC 41 kb)

## Data Availability

The datasets used and/or analyzed during the current study are available from the corresponding author on reasonable request.

## Abbreviations

ATB: Active tuberculosis
BCG: Bacillus Calmette-Guerin vaccination
BMI: Body mass index
CFP-10: Culture filtrate protein 10 kDa
DNA: Deoxyribonucleic acid
ESAT-6: Early secreted antigenic target 6 kDa
HIV: Human immunodeficiency virus
IFN-γ: Released γ-interferon
IGRA: Interferon-gamma release assay
MTB: *Mycobacterium tuberculosis*
PBMCs: Peripheral blood mononuclear cells
PDW: Platelet distribution width
PLT: Platelet
RNA: Ribonucleic acid
SFCs: Spot-forming cells
SRCC: Spearman’s rho correlation coefficient
TB: Tuberculosis
Th1: Helper T cells
T-SPOT.*TB*: T cell spot test for tuberculosis infection
