# Supplementary material for: Quantitative investigation of factors relevant to the T cell spot test for tuberculosis infection in active tuberculosis
Source: BMC Infect Dis. 2019 Jul 29;19:673. doi: 10.1186/s12879-019-4310-y (PMC6664742; doi:10.1186/s12879-019-4310-y)
Supplement: Supplementary file 1 — Smear grading report standarda. (DOC 31 kb) [file 12879_2019_4310_MOESM1_ESM.doc]

| **Additional file 1.** Smear grading report standard^a^. |
| --- |
| Sputum smear  +: 1–9 bacteria/50 fields;  1+: 10–49 bacteria/50 fields;  2+: 1–9 bacteria/field;  3+: 10–90 bacteria/field;  4+: ≥ 100 bacteria/field.  At least 50 fields were observed for the 2+ reports and at least 20 fields were observed for 3+ and above results.  Sputum culture  +: the actual colony count was reported, as the bacterial colony growth was less than 1/4 of the slope surface area;  1+: bacterial colony growth accounted for 1/4 of the slope surface area;  2+: bacterial colony growth accounted for 1/2 of the slope surface area;  3+ bacterial colony growth accounted for 3/4 of the slope surface area;  4+: bacterial colony growth accounted for entire the slope surface area. |
| ^a^: Refers to *Diagnostic Criteria and Principles of Management of Infectious Pulmonary Tuberculosis*（GB15987-1995）*.* |
