# Supplementary material for: Quantitative investigation of factors relevant to the T cell spot test for tuberculosis infection in active tuberculosis
Source: BMC Infect Dis. 2019 Jul 29;19:673. doi: 10.1186/s12879-019-4310-y (PMC6664742; doi:10.1186/s12879-019-4310-y)
Supplement: Supplementary file 3 — T-SPOT.TB results in pulmonary tuberculosis and extrapulmonary tuberculosis patients (DOC 51 kb) [file 12879_2019_4310_MOESM3_ESM.doc]

| **Additional file 3.** T-SPOT.*TB* results in pulmonary tuberculosis and extrapulmonary tuberculosis patients | | | | | | | | |
| --- | --- | --- | --- | --- | --- | --- | --- | --- |
|  | T-SPOT.*TB* false negative | |  | T-SPOT.*TB* true positive | |  | Chi-squared ^a^ | *P* value |
|  | N | % (95% CI) |  | N | % (95% CI) |  |  |  |
| Total tuberculosis patients (n = 360) |  |  |  |  |  |  |  |  |
| ESAT-6 | 31 | 8.61 (5.70-11.52) |  | 329 | 91.39 (88.48-94.30) |  |  |  |
| CFP-10 | 36 | 10.00 (6.89-13.11) |  | 324 | 90.00 (86.89-93.11) |  |  |  |
| T-SPOT.*TB* | 12 | 3.33 (1.47-5.20) |  | 348 | 96.67 (94.80-98.53) |  |  |  |
| Pulmonary tuberculosis (n = 297) |  |  |  |  |  |  |  |  |
| ESAT-6 | 26 | 8.75 (5.52-11.99) |  | 271 | 91.25 (88.01-94.48) |  |  |  |
| CFP-10 | 29 | 9.76 (6.37-13.16) |  | 268 | 90.24 (86.84-93.63) |  |  |  |
| T-SPOT.*TB* | 8 | 2.69 (0.84-4.55) |  | 289 | 97.31 (95.45-99.16) |  |  |  |
| Extrapulmonary tuberculosis (n = 63) |  |  |  |  |  |  |  |  |
| ESAT-6 | 5 | 7.94 (1.07-14.80) |  | 58 | 92.06 (85.20-98.93) |  | 0.044 | 0.834 |
| CFP-10 | 7 | 11.11 (3.13-19.09) |  | 56 | 88.89 (80.91-96.87) |  | 0.105 | 0.746 |
| T-SPOT.*TB* | 4 | 6.35 (0.16-12.54) |  | 59 | 93.65 (87.46-99.84) |  | 1.170^b^ | 0.279 |
| ^a^: Comparisons were performed in false negative rate of T-SPOT.*TB* between the pulmonary tuberculosis group and extrapulmonary tuberculosis group.  ^b^: Yates` continuity correction for the Chi-squared test.  *CFP-10* Culture filtrate protein 10 kDa, *CI* Confidence interval, *ESAT-6* Early secreted antigenic target 6 kDa. | | | | | | | | |
