# Supplementary material for: Quantitative investigation of factors relevant to the T cell spot test for tuberculosis infection in active tuberculosis
Source: BMC Infect Dis. 2019 Jul 29;19:673. doi: 10.1186/s12879-019-4310-y (PMC6664742; doi:10.1186/s12879-019-4310-y)
Supplement: Supplementary file 4 — Comparison of different influencing factors among tuberculosis patients with negative and positive T-SPOT.TB (dichotomy and grade variables) (DOC 105 kb) [file 12879_2019_4310_MOESM4_ESM.doc]

| **Additional file 4.**  Comparison of different influencing factors among tuberculosis patients with negative and positive T-SPOT.*TB* (dichotomy and grade variables) | | | | | | |
| --- | --- | --- | --- | --- | --- | --- |
| Variable | T-SPOT.*TB* false negative |  | T-SPOT.*TB* true positive | Chi-squared ^a^ | *P* value | |
|  | N (%) |  | N (%) |  |  |  |
| Total | 12 (3.33) |  | 348 (96.67) |  |  | |
| Sex |  |  |  | 0.009 | 0.925 | |
| Female | 2 (16.67) |  | 77 (22.13) |  |  | |
| Male | 10 (83.33) |  | 271 (77.87) |  |  | |
| Smoking^b^ |  |  |  | 2.055 | 0.152 | |
| No | 2 (16.67) |  | 145 (41.67) |  |  | |
| Yes | 10 (83.33) |  | 203 (58.33) |  |  | |
| Dust exposure^b^ |  |  |  | 1.578 | 0.209 | |
| No | 11 (91.67) |  | 246 (70.69) |  |  | |
| Yes | 1(8.33) |  | 102 (29.31) |  |  | |
| Previously treated cases |  |  |  | 0.000 | 1.000 | |
| No^c^ | 9 (75.00) |  | 248 (71.26) |  |  | |
| Yes^d^ | 3 (25.00) |  | 100 (28.74) |  |  | |
| Cavitation |  |  |  | 1.267 | 0.260 | |
| No | 7 (58.33) |  | 132 (37.93) |  |  | |
| Yes | 5 (41.67) |  | 216 (62.07) |  |  | |
| Extrapulmonary tuberculosis |  |  |  | 1.170 | 0.279 | |
| No | 8 (66.67) |  | 289 (83.05) |  |  | |
| Yes | 4 (33.33) |  | 59 (16.95) |  |  | |
| Diabetes mellitus |  |  |  | 0.282 | 0.595 | |
| No | 10 (83.33) |  | 320 (91.95) |  |  | |
| Yes | 2 (16.67) |  | 28 (8.05) |  |  | |
| Drug-resistant tuberculosis |  |  |  | 0.373 | 0.542 | |
| No | 12 (100.00) |  | 315 (90.52) |  |  | |
| Yes | 0 (0.00) |  | 33 (9.48) |  |  | |
| Stage^a^ |  |  |  | 1.231 | 0.540 | |
| Minimal/Mild | 0 (0.00) |  | 2 (0.57) |  |  | |
| Moderate | 3 (25.00) |  | 140 (40.23) |  |  | |
| Advanced | 9 (75.00) |  | 206 (59.20) |  |  | |
| Grade^b^ |  |  |  | 2.784 | 0.733 | |
| DNA/RNA positive | 3 (25.00) |  | 77 (22.13) |  |  | |
| The number of colony | 0 (0.00) |  | 24 (6.89) |  |  | |
| 1+ | 2 (16.67) |  | 100 (28.74) |  |  | |
| 2+ | 3 (25.00) |  | 47 (13.51) |  |  | |
| 3+ | 3 (25.00) |  | 67 (19.25) |  |  | |
| 4+ | 1(8.33) |  | 33 (9.48) |  |  | |
| ^a^: Yates` continuity correction for the Chi-squared test.  ^b^: Smoking or dust exposure for at least 3 months before a diagnosis of pulmonary tuberculosis.  ^c^: New cases are defined as not starting anti-TB treatment or being on anti-TB treatment for ＜1 month.  ^d^: Previously treated cases are defined as those anti-TB treated ≥1 month in the past. | | | | | |  |
