# Supplementary material for: Quantitative investigation of factors relevant to the T cell spot test for tuberculosis infection in active tuberculosis
Source: BMC Infect Dis. 2019 Jul 29;19:673. doi: 10.1186/s12879-019-4310-y (PMC6664742; doi:10.1186/s12879-019-4310-y)
Supplement: Supplementary file 5 — Comparison of different influencing factors among tuberculosis patients with negative and positive T-SPOT.TB (numeric variables) (DOC 80 kb) [file 12879_2019_4310_MOESM5_ESM.doc]

| **Additional file 5.**  Comparison of different influencing factors among tuberculosis patients with negative and positive T-SPOT.*TB* (numeric variables) | | | | | | | |  |
| --- | --- | --- | --- | --- | --- | --- | --- | --- |
| Variable | T-SPOT.*TB* false negative (n = 12) | |  | T-SPOT.*TB* true positive (n = 348) | | Z | *P* value | |
|  | Missing data  N (%) | Observed value,  Median (IQR)^a^ |  | Missing data  N (%) | Observed value,  Median (IQR)^a^ |  |  |  |
| Age, years | 0 | 57.50 (53.50-68.00) |  | 0 | 52.00 (36.00-66.00) | 1753.00 | 0.344 | |
| Body mass index, kg/m^2^ | 0 | 17.680 (16.745-20.113) |  | 6 (1.72) | 19.133(17.578-21.259) | 2620.50 | 0.133 | |
| Duration of symptoms, days | 0 | 166.50 (30.00-365.00) |  | 0 | 90.00 (30.00-546.00) | 1956.50 | 0.710 | |
| Hemoglobin, g/L | 0 | 111.50 (87.75-123.75) |  | 0 | 118.00 (105.00-135.00) | 2633.00 | 0.124 | |
| Platelet, ×10^9^/L, | 0 | 233.50 (152.25-358.75) |  | 0 | 258.50 (191.00-320.75) | 2251.00 | 0.646 | |
| Platelet distribution width, fL | 0 | 15.65 (12.48-16.03) |  | 0 | 13.90 (11.70-16.20) | 1898.00 | 0.592 | |
| ESR, mm/H | 2 (16.67) | 77.00 (34.00-92.25) |  | 30 (8.62) | 49.00 (26.00-71.00) | 1503.00 | 0.099 | |
| Prealbumin, mg/L | 3 (25.00) | 54.00 (27.00-82.60) |  | 36 (10.34) | 134.00 (82.08-196.75) | 3174.50^*^ | 0.002 | |
| Albumin, g/L | 0 | 24.55 (20.85-31.30) |  | 1 (0.29) | 32.02 (27.85-36.52) | 3139.00^*^ | 0.003 | |
| Globulin, g/L | 1 (8.33) | 33.61 (28.76-40.15) |  | 4 (1.15) | 32.45 (28.89-36.13) | 1901.50 | 0.599 | |
| Albumin to globulin ratio | 1 (8.33) | 0.785 (0.588-0.855) |  | 4 (1.15) | 0.967 (0.813-1.208) | 3034.00^*^ | 0.008 | |
| Alpha-1 globulin, g/L | 3 (25.00) | 5.66 (4.63-6.52) |  | 37 (10.63) | 4.62 (3.58-5.76) | 1523.50 | 0.111 | |
| Alpha-2 globulin, g/L | 3 (25.00) | 7.95 (5.57-9.14) |  | 37 (10.63) | 7.49 (6.36-8.64) | 2117.00 | 0.935 | |
| Beta-1 globulin, g/L | 3 (25.00) | 3.33 (2.41-3.91) |  | 37 (10.63) | 3.72 (3.27-4.19) | 2646.50 | 0.115 | |
| Beta-2 globulin, g/L | 3 (25.00) | 3.89 (3.26-4.81) |  | 37 (10.63) | 3.57 (3.09-4.22) | 1723.00 | 0.303 | |
| Gamma globulin, g/L | 3 (25.00) | 12.48 (10.45-17.74) |  | 37 (10.63) | 12.42 (10.14-14.97) | 1977.50 | 0.755 | |
| CD4+ T lymphocytes, cells/μL | 1 (8.33) | 118 (76.00-261.00) |  | 88 (25.29) | 310.00 (209.00-451.25) | 3109.50^*^ | 0.004 | |
| CD8+ T lymphocytes, cells/μL | 1 (8.33) | 117 (64.00-195.00) |  | 88 (25.29) | 259.50 (168.75-382.00) | 3245.50^*^ | 0.001 | |
| CD4+ to CD8+ ratio | 1 (8.33) | 0.978 (0.693-2.137) |  | 88 (25.29) | 1.222 (0.895-1.677) | 2189.50 | 0.773 | |
| ^a^: Data are presented as the value of variable.  ^*^: Correlation is significant at the 0.01 level (2-tailed).  *ESR* Erythrocyte sedimentation rate, *IQR* Interquartile range. | | | | | | | |  |
