# Supplementary material for: Quantitative investigation of factors relevant to the T cell spot test for tuberculosis infection in active tuberculosis
Source: BMC Infect Dis. 2019 Jul 29;19:673. doi: 10.1186/s12879-019-4310-y (PMC6664742; doi:10.1186/s12879-019-4310-y)
Supplement: Supplementary file 6 — Results of T-SPOT.TB between the two extreme BMI (DOC 41 kb) [file 12879_2019_4310_MOESM6_ESM.doc]

| **Additional file 6.** Results of T-SPOT.*TB* between the two extreme BMI | | | | | | | |
| --- | --- | --- | --- | --- | --- | --- | --- |
|  | T-SPOT.*TB* false negative | |  | T-SPOT.*TB* true positive ^a^ | |  | *P* value ^b^ |
|  | N | % (95% CI) |  | N | % (95% CI) |  |  |
| Extreme under-weight |  |  |  |  |  |  |  |
| BMI ＜ 16.00 kg/m^2^ (n = 38) | 2 | 5.26 (-2.17-12.70) |  | 36 | 94.74 (87.30-102.17) |  | 0.376 |
| BMI ≥ 16.00 kg/m^2^ (n = 316) | 10 | 3.16 (1.22-5.11) |  | 306 | 96.84 (94.89-98.78) |  |  |
| Extreme over-weight |  |  |  |  |  |  |  |
| BMI ＜ 25.00 kg/m^2^ (n = 240) | 12 | 3.53 (1.56-5.50) |  | 328 | 96.47 (94.50-98.44) |  | 1.000 |
| BMI ≥ 25.00 kg/m^2^ (n = 14) | 0 | 0 |  | 14 | 100.00 |  |  |
| ^a^: The number of missing cases was 6.  ^b^: Fisher`s exact probabilities in 2×2 table.  *BMI* Body mass index. | | | | | | | |
